# Identification and characterization of a Relish-type NF-κB, DvRelish, in *Dermacentor variabilis* in response to *Rickettsia rickettsii* infection

**DOI:** 10.3389/fcimb.2024.1494450

**Published:** 2024-12-16

**Authors:** Chanida Fongsaran, Victoria I. Verhoeve, Krit Jirakanwisal, Emma K. Harris, Kevin R. Macaluso

**Affiliations:** Department of Pathobiological Sciences, School of Veterinary Medicine, Louisiana State University, Baton Rouge, LA, United States

**Keywords:** Relish, tick, *Rickettsia*, NF-κB, immune response

## Abstract

Ixodid ticks serve as hosts and transmission vectors for several obligate intracellular bacteria, including members of the spotted fever group (SFG) of *Rickettsia*. Although ticks generate an immune response to bacterial insults, many of the signaling molecules associated with the response and how they may contribute to vector competence for *Rickettsia* are undefined. In this study, we isolated a full-length *dvrelish* transcript from *Dermacentor variabilis*, which encoded a Relish-type NF-κB. The presence of a canonical Rel homology domain (RHD) consistent with NF-κB proteins suggested a role in tick immune response for DvRelish. The expression of DvRelish was confirmed in tick tissues and fluorescent microscopy of tick hemocytes indicated increased expression following infection with *Rickettsia* as compared to a non-tick-borne bacterial pathogen. To further determine the effect of *dvRelish* gene knockdown on rickettsial infection, we used RNA interference-mediated gene knockdown in *D. variabilis* and demonstrated that transcription of *dvRelish* was decreased after 24 h post-injection of siRNA. We then assessed the response of *D. variabilis* when exposed to *Rickettsia rickettsii* and determined that transcription of *dvRelish* was inversely associated with rickettsial loads at 48 h post-exposure. Further studies are required to broaden the understanding of differential immune responses in ticks to SFG *Rickettsia* infection and elucidate the role played by the arthropod immune system in vector competence.

## Introduction

Ixodid ticks cause harm to vertebrate hosts through blood feeding and the transmission of disease-causing agents including viruses, protozoan parasites, and bacteria. As Gram-negative, obligate intracellular bacteria, the spotted fever group (SFG) of *Rickettsia* infect ticks and can be transmitted vertically between life cycle stages and horizontally from feeding ticks to vertebrate hosts. The duality in transmission routes is unique to the tick–SFG *Rickettsia* relationship, and the route employed is thought to be guided by the pathogenic nature of the *Rickettsia* (Perlman et al., 2006). The American dog tick, *Dermacentor variabilis*, is a vector and reservoir of *Rickettsia rickettsii*, the etiologic agent of Rocky Mountain spotted fever (RMSF). In addition to potentially lethal infection of humans, *R. rickettsii* can be pathogenic to the tick vector. Deleterious effects of *R. rickettsii* infection in *D. variabilis* ticks including reduced feeding, fecundity, and molting success have been previously reported (Niebylski et al., 1999; Schumacher et al., 2016; Harris et al., 2017). A fitness phenotype has been associated with rickettsial infection in *D. variabilis*, yet as rickettsiae are transmitted vertically, the tick response to rickettsial infection and the survival of rickettsiae are balanced.

Similar to other eukaryotes, ticks encode the signaling pathways that regulate the innate immune response such as toll, immunodeficiency (IMD), and Janus kinase/signal transducers and activators of transcription (JAK-STAT) (Gulia-Nuss et al., 2016; Sonenshine and Macaluso, 2017). Through a process originally described in *Drosophila melanogaster*, the expression of the antimicrobial peptide (AMP) genes depends on two members of the nuclear factor-κB (NF-κB) family of inducible transactivators: Dorsal-related immunity factors (DIFs) and Relish. Relish is responsive to Gram-negative bacterial infections (Ferrandon et al., 2007). For ticks, evidence of immune signaling pathways, including NF-κB, were identified in *Ixodes scapularis* (Smith and Pal, 2014; Gulia-Nuss et al., 2016). Specifically, sequences for a toll-like receptor with leucine-rich repeats, a Dorsal-type NF-κB and its regulating partner Cactus, a Relish-type NF-κB, and Caspar, a negative regulator of the IMD pathway, are present (Smith and Pal, 2014). Shaw et al. (2017) showed that lipids derived from *Borrelia burgdorferi* and *Anaplasma* species stimulate the IMD pathway in *I. scapularis* and *Dermacentor andersoni*, ultimately controlling the bacterial load in the tick. Components of the immune regulatory pathways, including the transcription factor Relish, have been identified in other tick species, including *Rhipicephalus microplus*, and are responsive to tick infection with *Anaplasma marginale* (Capelli-Peixoto et al., 2017). While receptors for initiation of the IMD pathway have not been identified in ticks, the unfolded protein response and a CD36-like protein called croquemort are alternates by which the IMD pathway is induced in *I*. *scapularis* (Sidak-Loftis et al., 2022; O'Neal et al., 2023). While the response of tick vectors to transiently infecting bacterial pathogens has been examined, the response to vertically transmitted pathogens such as SFG *Rickettsia* remains unresolved.

To understand the immune response in ticks during SFG *Rickettsia* infection, this study identified and characterized Relish-type NF-κB in *D. variabilis*. The results indicate that *D. variabilis* respond to *R. rickettsii* infection through the expression and activation of a Relish-type NF-κB protein. To further characterize the function of Relish-type NF-κB gene in *D. variabilis*, RNAi-mediated gene silencing was employed and the impact on rickettsial infection was observed. This study demonstrates that Relish-type NF-κB is involved in *D. variabilis* tick response to *R. rickettsii* infection and deepens the understanding of the functional roles of Relish-type NF-κB during the tick immune response.

## Material and methods

### Identification of a partial *dvrelish* transcript using previously published high-throughput sequencing databases

For the purpose of identifying previously unidentified *D. variabilis* transcripts with homology to Relish-type NF-κB proteins, a homology cloning approach was designed. Conserved domain searches of previously sequenced high-throughput sequence datasets and rapid amplification of cDNA ends-PCR (RACE-PCR) were used. A Blast search of the Genbank databases using the characterized Relish amino acid sequences from *D. melanogaster* (accession: Q94527), *Aedes aegypti* (accession: Q8MV44), and *Carcinoscorpius rotundicauda* (accession: ABC75034) as the query sequence returned no previously sequenced and annotated NF-κB gene transcript or protein sequence for *D. variabilis*. Three 454 pyrosequencing databases from published studies were previously released to NCBI’s Sequence Read Archive (Jaworski et al., 2010; Bissinger et al., 2011; Sonenshine et al., 2011) and consisted of unannotated partial transcripts isolated and sequenced from uninfected *D. variabilis* tissues, and whole *D. variabilis* injected with Gram-negative and Gram-positive bacteria, fungi, and ticks infected with intracellular *Anaplasma phagocytophilum* via feeding on an infected animal (accessions: SRX001954, SRX001955, and SRX018179). The sequencing datasets were combined and served as a local database for Blast (v2.2.27). The presence of partial transcripts containing domains characteristic of Relish-type NF-κB proteins was identified using the following domain alignments from the Conserved Domain Database (NCBI): (1) Rel homology domains (RHDs) (cd07++884 RHD-n_Relish); (2) immunoglobulin/plexin/transcription factor (IPT) domains (cd01177 IPT_NFkappaB); and (3) ankyrin repeats (cd00204 ANK). A reverse position-specific Blast (RPS-Blast) was performed using conserved domain database alignments for each of the canonical domains described above as the query. Identified partial transcripts were then used for primer design, transcript isolation, and cDNA library synthesis using the SMARTer RACE 5′/3′ cDNA synthesis kit (Clontech).

### Infection of *D. variabilis* with *R. rickettsii* and sample preparation

A colony of *Rickettsia*-free *D. variabilis* was maintained on rats, guinea pigs, and rabbits, as previously described (Macaluso et al., 2001). *Rickettsia rickettsii* (str. Shelia Smith) were maintained and propagated in Vero cells with Dulbecco’s modified Eagle’s medium (DMEM) (Invitrogen) supplemented with 5% fetal bovine serum (Hyclone). Cells were maintained in a 34°C incubator with 5% CO_2_. For rickettsial isolation, bacteria were partially purified when cells were ≥80% infected (Sunyakumthorn et al., 2008). Cells were lifted from a single infected T-75 flask and lysed with 10 passages through a 27-gauge needle. The resultant lysate was then centrifuged for 10 min at 275 × *g* at 4°C. The supernatant, which contained rickettsiae, was then passed through a 2-μm filter to remove host cell debris. Bacterial viability was determined using the Baclight viability staining kit (Invitrogen). Rickettsiae were enumerated with a Petroff-Hausser bacterial counting chamber on a Leica fluorescent microscope. Enumerated rickettsiae (2.5 × 10^8^) were subsequently resuspended in 10 μL of sterile phosphate-buffered saline (PBS).

Unfed, virgin female *D. variabilis* were injected with *R. rickettsii* (str. Shelia Smith). Prior to injection with rickettsiae, ticks were surface-sterilized with 5-min incubations of 0.1% bleach and 70% ethanol, and three times with distilled water. The ticks were then immobilized with tape, dorsal side down and injected into the hemocoel cavity via the coxae of the third left leg. Five unfed adult females were injected with 2 μL of *R. rickettsii* solution with a 33-gauge Hamilton needle. Ticks were maintained in a humidified environmental chamber at 27°C. At 1 h post-exposure, ticks were removed from the incubator and dissected in sterile PBS. Additionally, five uninfected, surface-sterilized ticks were dissected. Salivary glands, gut, ovaries, and hemolymph from infected ticks, and separately tissues from uninfected ticks, were combined for RNA extraction in 300 μL of TRIzol reagent (ThermoFisher). Prior to RNA isolation, tissues were homogenized using a TissueLyzer and 3-mm stainless steel beads (QIAGEN) in a 1.7-mL microcentrifuge tube for 4 min at 25 Hz/s. RNA was isolated as per the manufacturer’s instructions and stored at −80°C until used. Total RNA (1 μg) was treated with 2 units of Turbo DNase (Ambion) before cleanup and concentration with the Clean and Concentrator-5 kit (Zymo). Total RNA was subsequently reverse-transcribed using the iScript cDNA synthesis kit, including no reverse transcriptase reactions to identify DNA contamination.

### RACE-cDNA library synthesis, RACE-PCR, cloning, and sequencing

Total RNA (1 µg) was used for 5′- and 3′-enriched RACE-cDNA library synthesis using the SMARTer RACE 5′/3′ kit (Clontech) as per the manufacturer’s protocols. Primers were designed using Primer3 (Koressaar and Remm, 2007; Untergasser et al., 2012) from a partial *D. variabilis* transcript identified through RPS-Blast with homology to Relish-type RHD and are listed in **Table 1**. Each specific primer was combined with the Universal Primer Mix (Clontech) that amplifies the 5′ or 3′ adaptor in each library for PCR amplification. Traditional PCR with an additional round of cycling was performed with each RACE-PCR library and the appropriate direction-specific and transcript-specific primer. RACE-PCR was performed using the Advantage cDNA PCR kit (Clontech) with 1 µL of each library as template in separate PCR reactions. The thermocycling conditions consisted of 1 cycle at 95°C for 10 min, amplification for 40 cycles with denaturing at 95°C for 30 s, annealing at 45°C for 1 min, and extension at 72°C for 3 min. A final extension was performed for 10 min at 72°C. For the additional rounds of PCR, 0.5 µL of the previous reaction was used as the template for the next reaction. PCR reactions were visualized with a 1.5% agarose gel (GenePure) and SYBRSafe DNA gel stain (Invitrogen). All bands amplified were cloned using the TOPO TA Cloning kit with pCR4-TOPO (Invitrogen) per the manufacturer’s instructions. Plasmids were isolated using the Fast Plasmid Mini kit (Eppendorf) according to the manufacturer’s instructions. Plasmid inserts were sequenced using the dye terminator method on an Applied Biosystems 3130 Genetic Analyzer in GeneLab at Louisiana State University (LSU). Inserts were analyzed with MacVector (v14.5.0) and aligned with the isolated partial transcript sequence derived from traditional PCR with Clustal W.

**Table 1:** Primers used for the isolation of full-length *dvrelish* transcript.

Primer name	Primer sequence (5′-3′)	Purpose	Reference
IPTDV_43F	TGCACATCTGACTCCTGGAA	Initial isolation	This study
IPTDV_233R	ACAAAGGCTGGAAAGCTCAG	Initial isolation	This study
IPTLeggo211>5′	GACTATGGCCACCTGATGGT	5′ RACE-PCR	This study
RelishLeggo1925>3′	TGCCTTGTGACCCTTCTGA	3′ RACE-PCR	This study
RelishLeggo1247>3′	TGCAAGGCGGATACTCTACC	Sequencing	This study
RelishLeggo1797>3′	TGCTGACCTTTCACTTGTGG	Sequencing	This study
RelishLeggo2358>3′	CGGTCAAAAGTGGTGGAAGT	Sequencing	This study

### Analysis of the isolated *dvrelish* transcript

The full-length transcript, *dvrelish*, was aligned to previously isolated Relish-type NF-κB transcript sequences in other model organisms including the fruit fly *D. melanogaster* (accession: Q94527), the mosquito *Aedes aegypti* (accession: Q8MV44), and the horseshoe crab *C. rotundicauda* (accession: ABC75034) using MacVector (v14.5.0). Nucleotide and translated amino acid alignments were used to determine percent identities. A conserved domain search was performed using the Conserved Domain Database (NCBI) to identify all domains present on the transcript. The Open Reading Frame Finder (NCBI) was used to determine the correct open reading frame of the transcript. The cNLS mapper (Kosugi et al., 2009) was used to determine the presence of a nuclear localization sequence.

### Anti-DvRelish peptide antibody production

The production of an anti-DvRelish peptide antibody was commercially produced by YenZym Antibodies. Briefly, two peptides from the RHD of dvrelish were chosen and linked to the keyhole limpet hemocyanin (KLH) carrier protein separately. The two peptides used for immunization were as follows: CESSTQQRKTYPT KLENYNTQ-amide (DvRelish amino acids 47–67) and CYRRKIESLQPSQEEQRQLQ-amide (DvRelish amino acids 131–149). These peptides were chosen as candidates because they were predicted to be both hydrophilic and expressed on the surface of the protein. A rabbit was immunized with the combination of the two KLH-conjugated peptides in Freund’s complete adjuvant. After 2 months, the rabbit was inoculated with a secondary booster of both peptides. Serum was collected and the specificity of the produced antibodies in serum was determined by enzyme-linked immunosorbent assay. Antibody was then purified by high-performance liquid chromatography and delivered from the company at 0.24 mg/mL of purified mono-specific polyclonal IgG.

### Detection of DvRelish in tick tissue lysate

Uninfected adult females were dissected and tissues were placed in RIPA buffer with Complete Mini EDTA-free protease inhibitor cocktail (Roche) and homogenized using a sonicating probe (Sonic Dismembranator, Fisher) with 25% amplitude for 5 s, five times, on ice. The protein concentration in the tissue lysate was quantified using the DC Assay (Bio-Rad) per the manufacturer’s instructions. Tick protein (25 μg per lane) was separated with a Mini-ProteanX 4%–15% Tris-Glycine mini-gel (Bio-Rad). Separated proteins were transferred onto a 0.45-μm pore nitrocellulose membrane using a Trans-Blot semi-dry transfer cell at 25 V for 25 min. The membrane was blocked with 5% BSA in tris-buffered saline with 0.5% Tween 20 (TBST). Primary anti-DvRelish antibody (1:100) in 5% BSA in TBST was followed by secondary donkey anti-rabbit Li-Cor 800CW antibody (1:15,000). Peptide competition was performed with anti-DvRelish antibody (1:100) supplemented with 1 μg of each peptide the antibody was raised against. Blots were visualized with a Li-Cor Odyssey imager.

To confirm the antibody specificity, unfed female *D. variabilis* whole tick tissue was separated by SDS-PAGE. Bands of interest identified concurrently by Western blot at 100 and 70 kDa were excised from a 6% Tris-glycine gel with a clean scalpel blade. Samples were digested prior to mass spectrometry analysis with porcine pancreas-derived trypsin (Sigma-Aldrich). Digested samples were submitted for MALDI-TOF/TOF mass spectrometry on a Bruker UltrafleXtreme MALDI-TOF/TOF MS system (Bruker Daltonics) at the LSU Chemistry Department. For identification of submitted samples, reported sample peptide masses were compared to predicted masses for the putative amino acid sequence of DvRelish. *In silico* trypsin digestion analysis of the putative DvRelish amino acid sequence was performed with one missed cleavage allowed using the PeptideMass predictor program from the Swiss Institute of Bioinformatics ExPAsy website (Wilkins et al., 1997).

### *DvRelish* RHD construction, transfection of HEK293T/17 cells, and peptide characterization

The insert fragment was amplified using the primers shown in **Table 2** to generate insert fragments containing the 5′-*Bgl*II and 3′-*BamH*I recognition sites, KOZAK sequence, and 6×His tag. The human embryonic kidney cell line HEK293T/17 (ATCC CRL-11268) was transfected with eukaryotic expression vectors (pAcGFP1-C1) (Clontech) harboring the RHD, using Lipofectamine 3000 (Invitrogen). Briefly, 4×10^6^ HEK293T/17 cells were seeded into 60-mm tissue culture dishes and maintained under standard conditions for 24 h. Mixtures of Lipofectamine 3000 (10 μL) and 5 μg of pAcGFP1-C1 with/without truncated *DvRelish* plasmids in Opti-MEM Reduced Serum Medium (Invitrogen) were incubated at room temperature for 15 min and then added onto HEK293T/17 culture plates. After 20 h post-transfection, the culture medium was replaced with fresh medium and transfected cells were collected at 48 h after transfection. Subsequently, the culture medium was removed, and cells were washed twice with 1× PBS. For 60-mm tissue culture dishes, 500 μL of lysis buffer (50 mM Tris-HCl, pH 7.4, 150 mM NaCl, and 1% Nonidet P-40) containing protease inhibitor cocktail (Bio Basic Inc.) was added and the cells were harvested and transferred to a 1.5-mL microcentrifuge tube. The collected cells were incubated on ice for 15 min and then the cell debris was removed by centrifugation at 2,500 × *g*, 4°C for 15 min. Total protein was collected from the supernatant and transferred to a new microcentrifuge tube. The proteins were applied to a Capturem™ His-Tagged Purification Miniprep column (Takara) and performed according to the manufacturer’s instructions. Recombinant DvRelish expression was analyzed via SDS-PAGE and Western blot. SDS-PAGE was performed using 4%–12% Mini-PROTEAN^®^ TGX™ Precast Protein Gels (Bio-Rad). Then, the protein was transferred to 0.45 μM pore nitrocellulose (Bio-Rad) using a Trans-Blot SD semi-dry transfer apparatus (Bio-Rad). For Western blot analysis, the membranes were incubated with a mouse monoclonal 6× anti-His monoclonal antibody (1:5,000; Takara, Clontech) or a rabbit polyclonal anti-DvRelish peptide antibody (1:1,000; Yenzyme), followed by a secondary antibody of donkey anti-mouse Li-Cor 680 CW (1:20,000; Li-Cor) or a donkey anti-rabbit Li-Cor 800 CW antibody (1:15,000). Western blots were imaged using a Li-Cor Odyssey imager (Li-Cor).

**Table 2:** Primers used to construct the DvRelish RHD for gene expression analysis and siRNA generation.

Primer name	Primer sequence (5′-3′)	Purpose	Reference
NheI-RHD_F^a,b^	cta **gctagc** cgccaccatgcaccatcaccatcaccatATGCCTATC	RHD construction	This study
BamHI-IPT_R^a,b^	cgc **ggatcc** ttaAGGCAGATAGGTGAACTCAA	RHD construction	This study
siDvRelish2_Antisense^c^	aaGACTGCTGTTTTGTTACAGcctgtctc	siRNA	This study
siDvRelish2_Sense^c^	aaCTGTAACAAAACAGCAGTCcctgtctc	siRNA	This study
siGFP_Antisense^c^	aaATTTTCTGTCAGTGGAGAGcctgtctc	siRNA	This study
siGFP_Sense^c^	aaCTCTCCACTGACAGAAAATcctgtctc	siRNA	This study

a Engineered restriction enzyme sites are highlighted in bold text and underlined.

b Lowercase: vector sequence, upper case: insert sequence in pAcGFP-C1.

c Uppercase: target sequence.

### Electrophoretic mobility shift assay of recombinant *dvRelish* RHD

Recombinant *DvRelish* RHD in HEK293T cells were resuspended in 500 μL of cell lysis buffer (20 mM Tris, pH 7.4, 150 mM NaCl, 1% Nonidet P-40, 0.5% sodium deoxycholate, and 0.1% SDS) containing protease inhibitors. The cell suspensions were lysed in an ice-water bath during sonication for 5 min. The lysate was centrifuged at 12,000 × *g* for 10 min at 4°C, and the pellet was discarded. Electrophoretic mobility shift assay (EMSA) was performed according to the manufacturer’s instructions from Odyssey EMSA Kit (LI-COR). Briefly, 10 μg of r*DvRelish* RHD proteins was incubated with 10× Binding buffer, 1 μg/μL Poly(dI·dc), 25 mM DTT, and IRDye 700 NF-κB oligonucleotide. The mixture was incubated at room temperature for 30 min in the dark. The sample was then mixed with 10× Orange loading dye and loaded onto 4% Native gels for electrophoresis. To evaluate the binding, the gel was imaged with the Odyssey Infrared Imaging System.

### DvRelish expression in hemocyte samples for fluorescent microscopy

Bacteria were used to assess DvRelish expression in tick hemocytes. *R. rickettsii* was purified via sucrose gradient. Briefly, infected cells were lysed by repeated passage through a needle, followed by centrifugation to separate *Rickettsia* over a 20% sucrose cushion and kept at −80°C in SPG buffer (218 mM sucrose, 3.8 mM KH_2_PO_4_, 7.2 mM K_2_HPO_4_, and 4.9 mM L-glutamate, pH 7.2) (Chan et al., 2011). Total concentration of rickettsiae was quantified via endpoint dilution assay as previously described (Esteves et al., 2020). Overnight cultures of *Pseudomonas aeruginosa* (ATCC 25873) were grown in a 37°C shaking incubator in trypic soy broth (Invitrogen) and assessed for viability and enumerated using the Baclight viability staining kit, as described previously. Bacteria were pelleted via centrifugation at 4°C for 10 min at 16,000 × *g* and resuspended in sterile PBS.

Five unfed adult *D. variabilis* female ticks per group were injected 10^7^ P*. aeruginosa* (ATCC 27853) or 10^7^ *R. rickettsii*. The ticks were surface-sterilized and immobilized dorsal side-down using tape and injected as mentioned previously. After 1 h, hemolymph was collected from the coxal–trochanteral joint and smeared on a glass slide. The slide was air-dried, fixed in 4% paraformaldehyde with 4% sucrose in PBS for 10 min, washed with PBS, permeabilized with 0.1% Triton X-100 in PBS for 15 min, blocked with 3% BSA in PBS for 1 h, and then washed twice with 0.03% Triton X-100 in PBS. Hemocytes were incubated with anti-DvRelish peptide antibody (1:50) at 4°C for 1 h. Following incubation, the cells were washed three times and then incubated with an FITC-conjugated goat anti-rabbit antibody (1:100) for 1 h at room temperature. Samples incubated without primary anti-DvRelish antibodies serve as a control for off-target secondary antibody binding. After the final wash, the cells were mounted with VECTASHIELD mounting medium containing DAPI (Vector Laboratories) and examined using an Olympus FLUOVIEW confocal microscope.

### Knockdown and expression of *dvRelish* and quantitative reverse transcription PCR

siRNA templates were designed from target sites on DvRelish (Genbank accession number KJ484815.1) and the green fluorescent protein (Genbank accession number U62637.1). The sequences were subjected to siRNA template design to generate DNA oligonucleotides sequences for the Silencer^®^ siRNA Construction Kit (Ambion). siRNA templates were synthesized following the Silencer^®^ siRNA Construction Kit (Ambion) protocol and the concentrations were determined using a NanoDrop^®^ ND-1000 UV-Vis Spectrophotometer (Thermo Fisher Scientific).

For microinjection, five unfed adult female ticks were surface-sterilized and immobilized as previously described. Each tick was injected with 1 μL of PBS containing either 250 or 500 ng of siRNA using a 33-gauge Hamilton needle and a 5-μL glass syringe into the coxae of the third left leg. Ticks were placed in a humidified incubator set at 27°C under 90% humidity for 24 h. The following day, the surviving ticks were injected with 1 µL containing 10^7^ bacterial suspension using a 33-gauge Hamilton needle and a 5-µL glass syringe into the coxae of the third left leg. Ticks were incubated in a 27°C humidified environmental chamber for 12, 24, and 48 h post-exposure.

Total RNA was extracted from adult *D. variabilis* tissues (combined: salivary glands, gut, ovary, and hemolymph) using TRIzol reagent (Molecular Research Center) following standard protocols. Following quantification of RNA via a NanoDrop ND-1000 UV-Vis Spectrophotometer, 2 units of TurboDNase (Invitrogen) was added and incubated at 37°C for 30 min. RNA was purified and concentrated using the Clean and Concentrator-5 Kit (Zymo Research) and eluted in 20 μL of DNase/RNase-free water. Total purified RNA (250 ng) was used with the iScript™ cDNA Synthesis Kit (Bio-Rad). No reverse transcriptase reactions (water was added instead of reverse transcriptase) were performed to assess residual DNA by qPCR.

Gene expression was quantified by qPCR on a LightCycler (Roche). The amplification reaction was performed in triplicate in a final volume of 35 μL using 10 μM of specific primers and probes (**Table 3**) and 5 μL of cDNA as template and iTaq Universal Probes Master Mix (Bio-Rad). Ten microliters of the qPCR reaction mix was assessed in triplicate on a 384-well plate and amplified using a Light Cycler 480 Real-Time PCR system (Roche). The qPCR conditions consisted of an initial cycle at 95°C for 3 min for denaturation, followed by 45 cycles at 95°C for 15 s for denaturation, 60°C for 1 min for annealing, and 72°C for 1 s for extension, followed by cooling down to 40°C for 30 s. Serial dilutions of the pCR4-TOPO plasmid containing amplicons for each primer set genes were assessed in parallel, serving as a standard for concentration analysis. Serial diluted standards with known copy numbers, as well as environmental and negative (PCR water) controls, were included along with experimental samples for each set of reactions.

**Table 3:** Primers and probes for qPCR.

Primer name	Primer sequence (5′-3′)	Reference
DvRelish-187_Fw	AACTACAATACACAGTTACCCCAT	This study
DvRelish-293_Rv	ACTGTAACAAAACAGCAGTCTT	This study
DvRelish-HEX	HEX/TC ATG TCC A/ZEN/T CGT ATC ACC ATG CGA/3IABkFQ/	This study
DvActin-1424For	CTTTGTTTTCCCGAGCAGAG	Sunyakumthorn et al. (2012)
DvActin-1572Rev	CCAGGGCAGTAGAAGACGAG	Sunyakumthorn et al. (2012)
DvActin-Cy5	Cy5/TC ATG TCC A/TAO/T CGT ATC ACC ATG CGA/3IAbRQSp/	This study
ompBRr1370F	ATAACCCAAGACTCAAACTTTGGTA	Jiang et al. (2005)
ompBRr1494r	GCAGTGTTACCGGGATTGCT	Jiang et al. (2005)
RrompB-FAM	FAM/CGCGATCTTAAAGTTCCTAATGCTATAACCCTTACCG ATCGCG	Harris et al. (2017)

### Statistical analysis

All data are presented as mean ± standard error of the mean (SEM). Statistical significance between two groups was determined by *t*-test with a *p*-value ≤ 0.05 considered significant. Additionally, comparative differences among multiple experimental groups were analyzed by analysis of variance (ANOVA), followed by the Tukey’s *post-hoc* test with a *p*-value ≤ 0.05 considered significant. All statistical analyses were performed using the GraphPad Prism Software.

## Results

### Isolation of a partial *dvrelish* transcript and completion of full-length transcript via RACE-PCR

The RPS-Blast of the *D. variabilis* 454 pyrosequencing database resulted in one partial transcript with an RHD, one partial transcript with an IPT domain, and two partial transcripts with ankyrin repeats. The sequences for RHD-containing and IPT-containing transcripts were utilized for primer design for PCR. Both primer sets were used in traditional PCR with cDNA from uninfected ticks and *D. variabilis* infected with *R. rickettsii.* Resultant amplicons were cloned and sequenced. No partial transcripts with homology to previously identified Relish-type NF-κB were identified in cDNA libraries from uninfected *D. variabilis*. Traditional PCR using cDNA from *R. rickettsii*-infected *D. variabilis* as template resulted in the amplification of two partial transcripts with identity to known NF-κB transcripts. However, only the primer set specific for IPT domains was successful in amplifying the intended target whereas the RHD-specific primers instead amplified an alternative RHD characteristic of another RHD-containing NF-κB protein, Dorsal. Primers for RACE-PCR with both 5′ and 3′ enriched libraries were then designed with at least 100 nucleotides to overlap RACE-PCR amplicons with traditional PCR amplicons. These primers were paired with the universal primer mix (UPM) primers specific to the 5′ or 3′ adaptor, which was ligated during the RACE-library preparation. Amplicons were not immediately visualized with 40 cycles of PCR; thus, 0.5 µL of first-round reactions was used as the template for a second round of 40 cycles of PCR. All amplicons visualized were cloned into pCR4-TOPO and sequenced. Amplification of the 5′-end of the *dvrelish* transcript occurred with additional rounds of RACE-PCR with primer IPTLeggo211>5′. A single band overlapped with the known partial transcript after sequencing with M13 Forward and M13 Reverse primers and completed the 5′ sequencing of the *dvrelish* transcript. Amplification of the 3′-end of *dvrelish* transcript occurred with RelishLeggo1925>3′ and the UPM with additional rounds of RACE-PCR and sequencing of all amplicons. One large amplicon of approximately 2,500 base pairs (bp) overlapped with the previously known sequence. Complete sequencing of the cloned amplicon was performed with primer walking. Primers RelishLeggo1247>3′, RelishLeggo1797>3′, and RelishLeggo2358>3′ were used for the sequencing, completing the 3′-end of the transcript. The full-length transcript was deposited into Genbank under the accession KJ484815.

### Analysis of isolated *dvrelish* transcript

**Figure 1:**
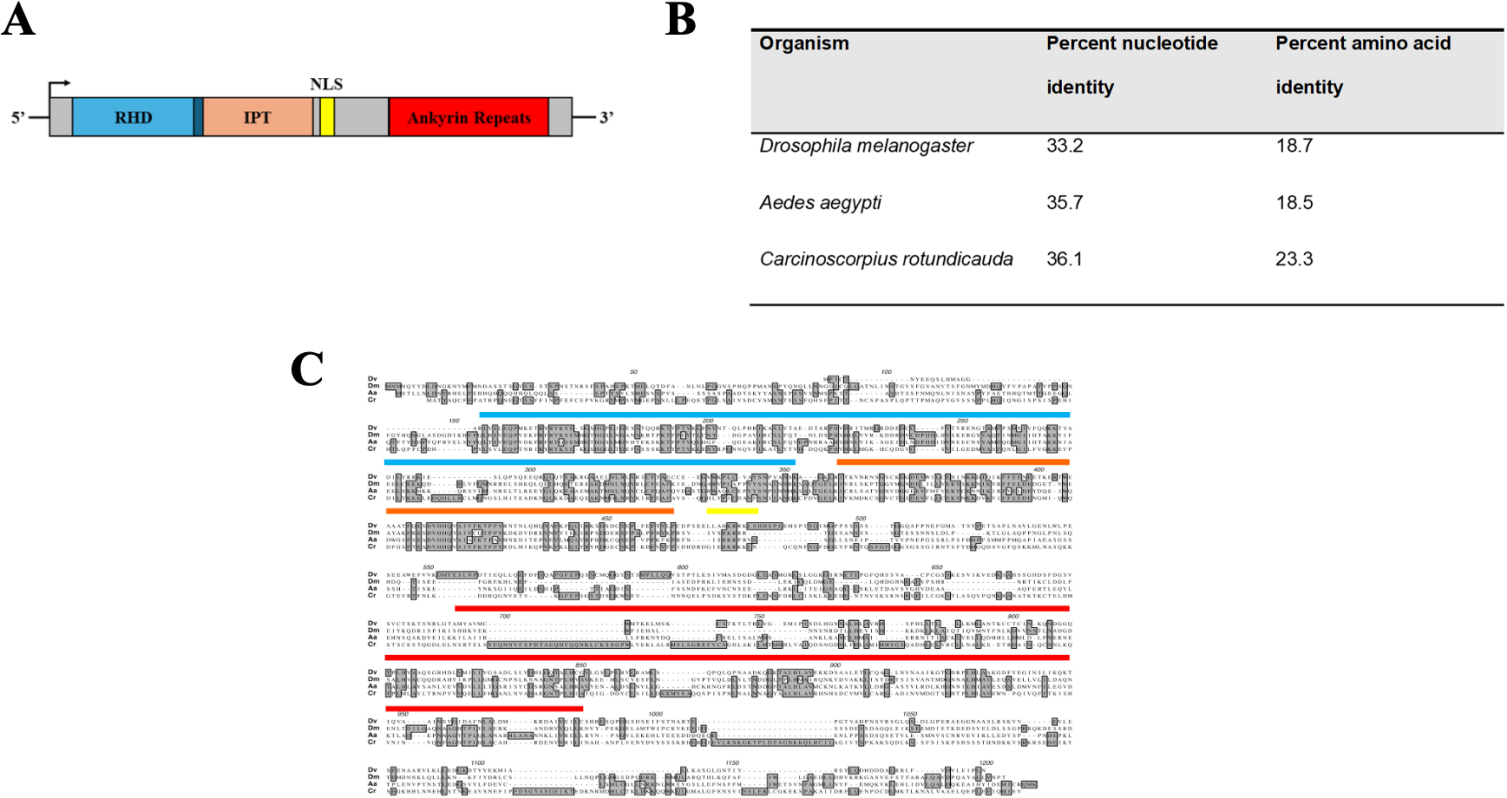
**(A)** Schematic representation of the *dvrelish* transcript. RHD represents the Rel homology domain; IPT represents the immunoglobulin, plexin, transcription factor domain; and NLS represents nuclear localization sequence. **(B)** Percent identity of isolated *dvrelish* transcript and putative translated DvRelish protein as compared to the Relish-type NF-κB of other model organisms. **(C)** Multiple sequence comparison of Relish-type NF-κB translated amino acid sequence. *dvrelish* was translated and aligned to Relish-type NF-κB molecules of *Drosophila melanogaster* (accession: Q94527), *Aedes aegypti* (accession: Q8MV44), and *Carcinoscorpus rotundicauda* (accession: ABC75034). The conserved domains are represented by boxes on the alignment. Specifically, the Rel homology domain is labeled from amino acid (AA) 156 to 351, the IPT domain is labeled from AA 361 to 463, the nuclear localization sequence is labeled from AA 470 to 479, and the ankyrin repeat domain is labeled from AA 689 to 983. Shading with the amino acid alignment indicates identities across the aligned sequences. DBS represents regions corresponding to DNA binding sites. ABS represents regions corresponding to ankyrin binding sites.

A schematic representing the domain architecture was determined through searches with the Conserved Domain Database, ORF finder, and cNLS mapper for the putative-translated transcript and is presented in **Figure 1A**. The full-length *dvrelish* transcript was 3,138 nucleotides in length with an ORF that starts at base 409 through the stop codon beginning at base 3031. The putative translated ORF is 873 amino acids long. The conserved domain search determined the presence of a RHD (amino acids 20–193), an IPT domain (amino acids 197–300), a nuclear localization sequence (amino acids 307–317), and five ankyrin repeats (amino acids 520–751).

The percent identities for the nucleotide alignment and translated amino acid alignment are listed in **Figure 1B**. In general, the nucleotides align slightly better than the amino acid sequences. The closest nucleotide and amino acid sequence were from the horseshoe crab, *C. rotundicauda*, with nucleotide and amino acid identities of 36.1% and 23.3%, respectively. Compared to the *I. scapularis* p105-like transcript (ISCW018935), which does not contain the canonical inhibitory ankyrin repeats, there is 58.4% nucleotide identity, and 35.8% translated amino acid identity across the conserved regions. The translated amino acid sequence of the transcript aligned to Relish-type NF-κB proteins of other model organisms. The nucleotide sequence is minimally conserved with other arthropods, including the mosquito vector *A. aegypti*. The transcript sequence encoding the *Drosophila* NF-κB contains multiple stretches of nucleotides within the RHD and IPT domains that are not encoded in the mosquito or horseshoe crab Relish NF-κB transcripts. Additionally, the horseshoe crab Relish-type NF-κB transcript encodes numerous additional stretches of nucleotides present in the C-terminal ankyrin repeat domains that were previously identified as linker sequence (Fan et al., 2008). In stark contrast, the transcript encoding *dvrelish* contains only two linker sequences.

Interestingly, while the putative translated amino acid sequence of *dvrelish* has minimal amino acid identity to other arthropods, the conserved domain search reveals that the RHD, IPT, and ankyrin repeats are highly conserved (**Figure 1C**). The recognized domains and their specific amino acid sequence correspond to structures that are integral to the function of Relish-type NF-κB proteins. DNA binding sites and ankyrin repeat binding sites throughout the RHD and IPT were conserved in DvRelish.

### Detection of DvRelish in tick tissue lysate

**Figure 2:**
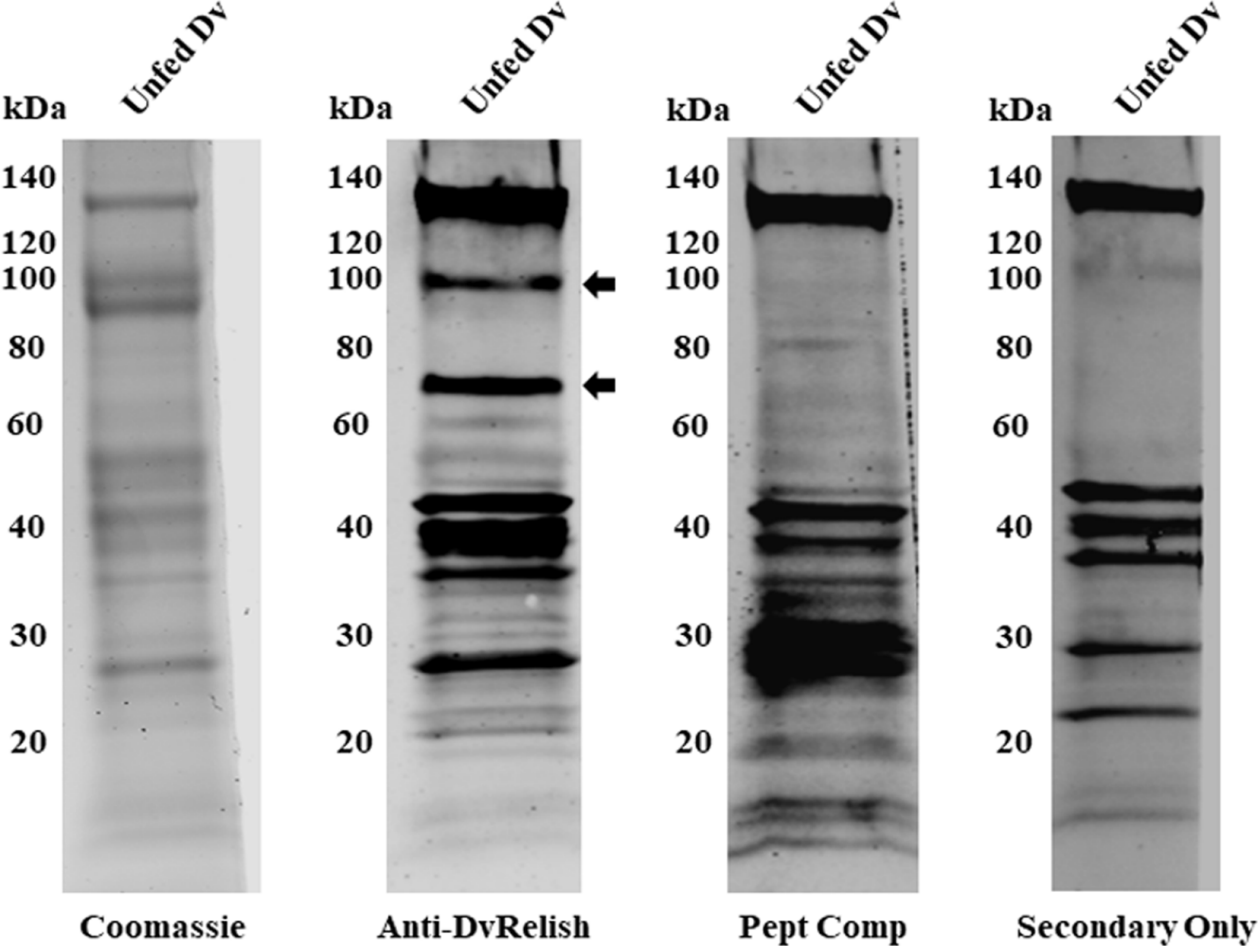
Expression of DvRelish in *D. variabilis* tissues. Expression of DvRelish in 25 µg of protein from unfed adult female *D. variabilis* tissue lysate. DvRelish was recognized via Western blot with anti-DvRelish antibody. A peptide competition assay (Pept Comp) decreased recognition of proteins at 100 and 70 kDa.

DvRelish expression was queried in *D. variabilis* tissues. All tissues of the tick were homogenized and centrifuged to remove insoluble tick materials. Tick protein (25 µg) was probed via Western blot with anti-DvRelish antibody, resulting in the recognition of multiple protein bands (**Figure 2**). A peptide competition assay was performed to determine the specificity of anti-DvRelish binding. The addition of 1 µg of each peptide to the primary DvRelish incubation resulted in the loss of signal at 100 and 70 kDa. The secondary antibody only control was performed, yielding non-specific binding of the donkey anti-rabbit 800CW antibody to tick tissues. Despite non-specific binding to *D. variabilis* proteins, the peptide competition assay demonstrated that the anti-DvRelish antibody recognized two proteins at 100 and 70 kDa. Both proteins were identified by mass spectrometry and the resultant peptide masses were compared to the masses predicted for DvRelish by *in silico* trypsin analysis. The predicted DvRelish peptide masses corresponded with 18 identified peptide masses and with 9 peptide masses identified within the 70-kDa protein. Additionally, the 70-kDa protein masses identified by mass spectrometry corresponded with only three predicted masses with a translated partial putative *dvdorsal* transcript corresponding to a Dorsal-type NF-κB protein previously isolated. Together, these data confirm the recognition of DvRelish via the anti-DvRelish antibody at 100 kDa and the N-terminal DvRelish at 70 kDa.

### rDvRelish expression and purification

**Figure 3:**
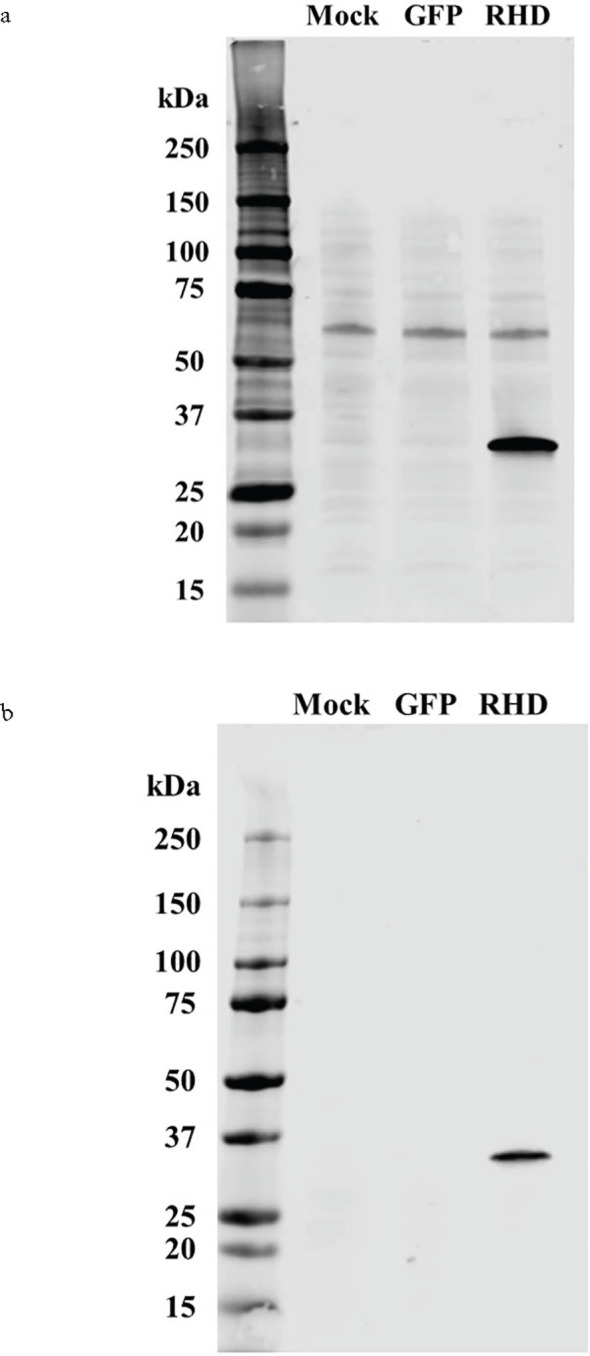
Western blot analysis of recombinant expression and protein purification of DvRelish. The rDvRelish expression plasmid with N-terminal 6×His tag was used to transfect HEK293T cells. The lysates were resolved on a 4%–12% SDS-PAGE gel. Lane 1 contained lysate from cells transfected without the vector plasmid (mock). Lane 2 contained lysate from cells transfected with the vector plasmid (pAcGFP-C1). Lane 3 contained rDvRelish RHD protein with 6×His tag. The membrane was probed with anti-His antibodies **(A)** and customized antibodies targeting DvRelish peptides **(B)**.

For EMSA, the DvRelish RHD was cloned in vector plasmid (pAcGFP-C1) with N-terminal 6×His tag. The DvRelish expression plasmid was transfected into HEK293T cells using Lipofectamine 3000. The transfected cells were detected with fluorescence microscopy (**Supplementary Figure 1**) and total protein lysates were collected and purified by His-tagged column, followed by Western blot analysis. The recombinant protein was detected with both anti-His (**Figure 3A**) and anti-DvRelish (**Figure 3B**) antibodies. These results suggest that the recombinant DvRelish protein is expressed.

### Electrophoretic mobility shift assay

**Figure 4:**
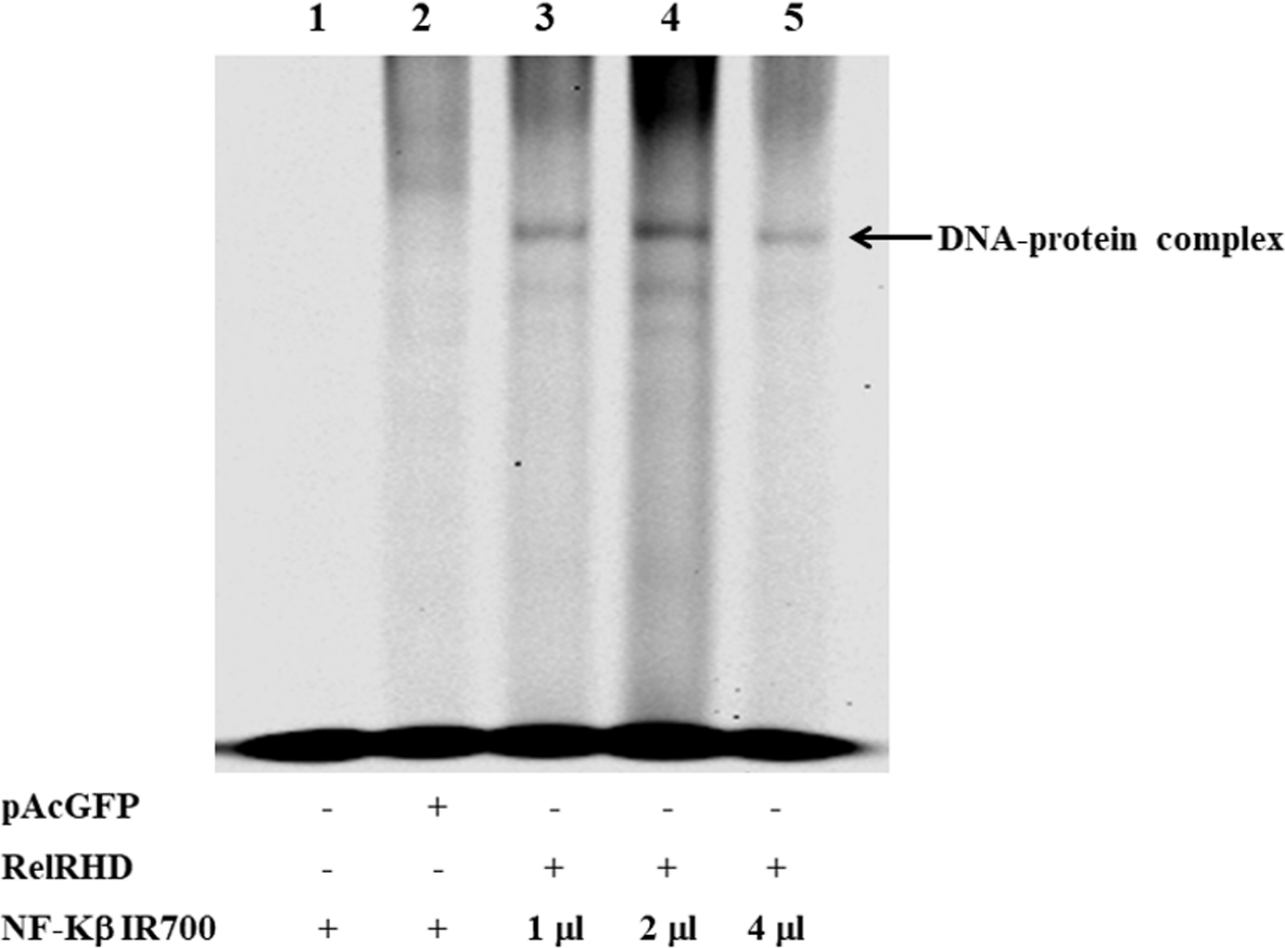
EMSA analysis demonstrating recombinant DvRelish RHD binding to kB motifs. EMSA was performed using 5′IR700 consensus NF-κB probes to assess binding specificity. The gel image shows that the recombinant protein specifically binds to κB motifs. Lanes 1 and 2 serve as control for non-specific binding. Lanes 3, 4, and 5 show rDvRelish RHD binding to κB motifs 50, 100, and 200 fmol, respectively. The arrowhead indicates the DNA–protein complex.

We further characterized DvRelish via EMSA. Recombinantly expressed DvRelish RHD was incubated with fluorescently labeled NF-κB consensus DNA motif probes. In the control experiments, no protein/DNA complex or only pAcGFP-C1 vector (**Figure 4**, lanes 1-2), nonspecific binding was not observed. The EMSA results showed that the DvRelish RHD could bind to the NF-κB DNA motifs (**Figure 4** lanes 3–5), demonstrating the DNA-binding capacity of the transfected Relish protein. This suggests that the recombinant DvRelish RHD has an interaction with NF-κB motif. DvRelish could function as a transcription factor to activate κB motifs and regulate the expression of immune-related genes in *D. variabilis*.

### Immunofluorescence assay of *D. variabilis* hemocytes

**Figure 5:**
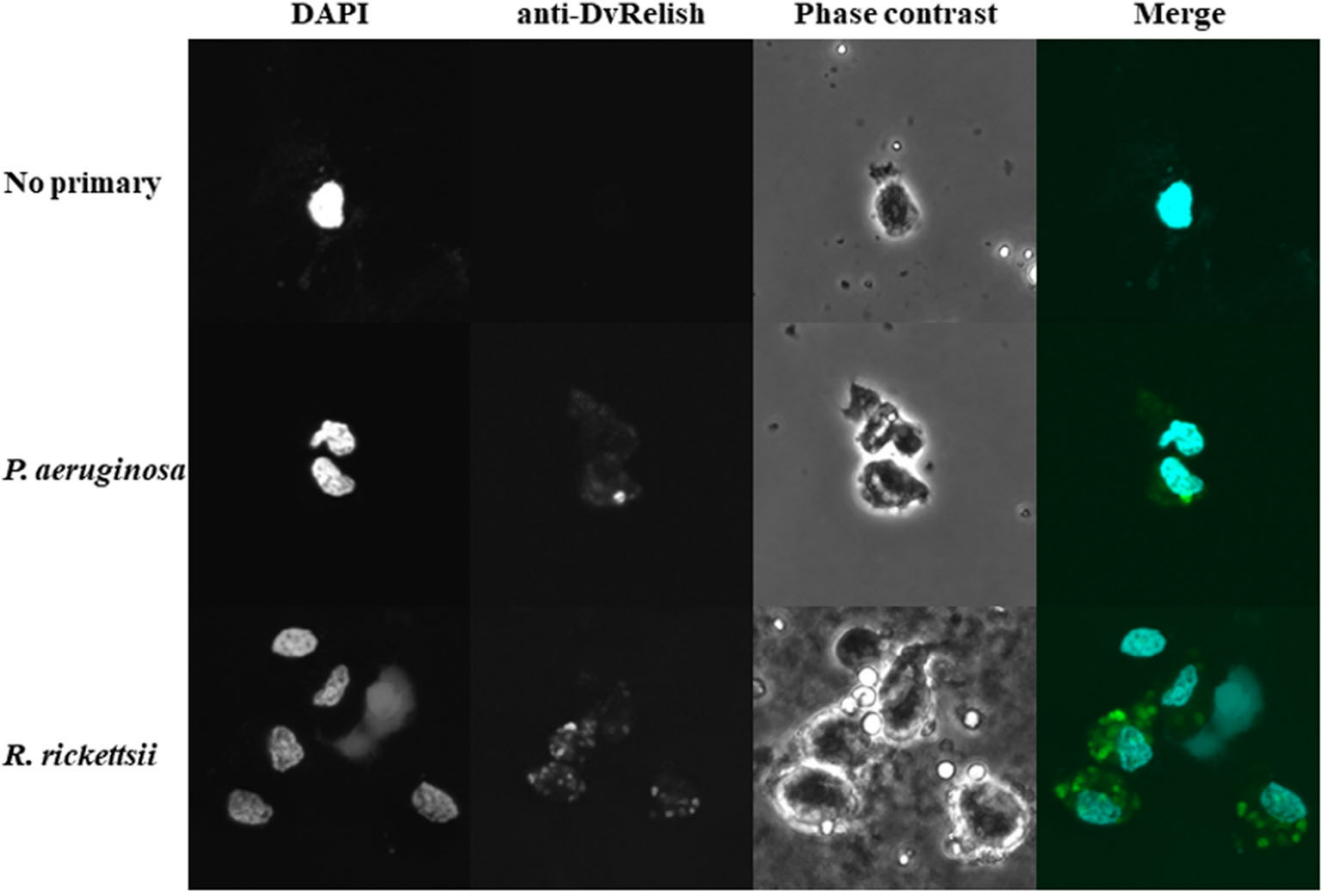
Immunofluorescence detection of tick hemocytes. Expression of DvRelish and nuclear localization in response to injected bacteria. Adult, unfed *D. variabilis* females were exposed to *R. rickettsii* or *P. aeruginosa*. After 1 h of exposure, hemolymph was collected, and hemocytes were strained with DAPI to visualize nuclear localization. DvRelish expression was detected using an anti-rabbit FITC-conjugated secondary antibody.

Because hemocytes are an important site of AMP production, the hemocytes of unfed *D. variabilis* females were collected and spotted onto slides for IFA to detect DvRelish expression and nuclear localization in the presence of bacterial infection. To further explore the response of *D. variabilis* hemocytes to *R. rickettsii*, adult unfed *D. variabilis* females were injected with either 10^7^ *R. rickettsii* or *P. aeruginosa*. Following exposure for 1 h, the hemolymph was collected, and the cells were stained with anti-DvRelish as previously described. DvRelish was present within the cytoplasm of the hemocytes in both *R. rickettsii*-injected and *P. aeruginosa*-injected but not in control hemocytes (**Figure 5**). While qualitative, the intensity of *R. rickettsii* treatment is greater than that observed in the *P. aeruginosa*-treated hemocytes.

### Functional knockdown of DvRelish via RNA-mediated interference

**Figure 6:**
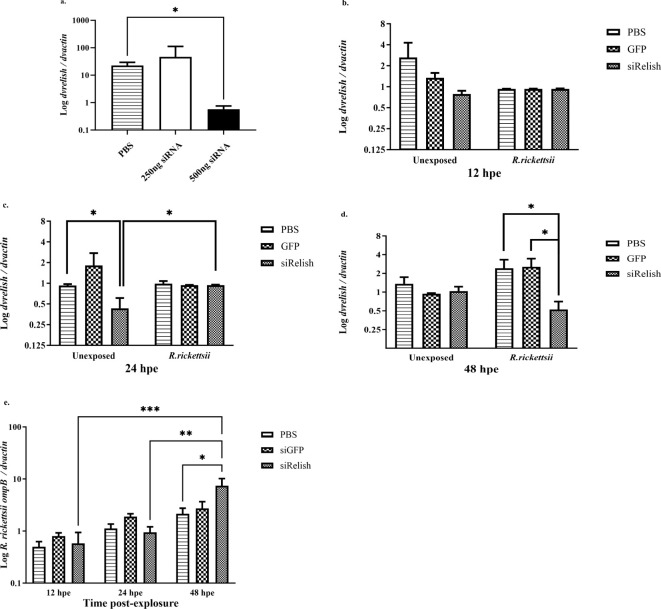
**(A)** Expression of DvRelish mRNA in *D. variabilis* ticks after Relish gene silencing. DvRelish was successfully knocked down using 500 ng of siRNA. **(B–D)** With siRNA, DvRelish mRNA expression was measured in unexposed ticks and *R. rickettsii* exposed ticks at 12, 24, and 48 h post-exposure. The transcription level of the *dvrelish* gene was determined by RT-qPCR and normalized against *dvactin*. **(E)** *R. rickettsii ompB* mRNA levels were assessed after gene knockdown by RT-qPCR and normalized against *dvactin*. Data are presented as mean relative expression, with error bars representing the standard error of the mean (SEM). Statistical significance for DvRelish gene silencing was determined using a *t*-test, with *p*-values ≤ 0.05 considered significant. Statistical significance for siRNA DvRelish in unexposed and *R. rickettsii*-exposed ticks was determined using a two-way ANOVA with Tukey’s *post-hoc* test, with a *p*-value ≤ 0.05 considered significant. *p < 0.05, **p < 0.01, and ***p < 0.001.

We sought to determine whether expression of *dvrelish* in *D. variabilis* ticks could be downregulated via siRNA-mediated gene silencing. Adult, unfed *D. variabilis* females were injected with 250 ng, 500 ng, or PBS as a control and incubated for 24 h. The expression of DvRelish mRNA was analyzed by qPCR. The results showed that there was reduced expression of DvRelish transcript with 500 ng of siRNA at 24 h post-injection (**Figure 6A**).

To determine the effect of the reduced expression of DvRelish during the tick response to *R. rickettsii* infection, adult, unfed *D. variabilis* females were injected 500 ng of siRNAs directed against *dvrelish*, GFP, or PBS as a control and incubated for 24 h. Ticks were then injected with 10^7^ *R. rickettsii* or PBS. Following exposure for 12, 24, and 48 h, ticks were collected to determine the expression of *dvRelish*. Expression of DvRelish mRNA was determined by qPCR and normalized to *dvactin* at the selected time points (**Figures 6B–D**). The results showed that the transcriptional expression levels were not significantly different at 12 hpe, and exposure to *R. rickettsii* did not impact the transcription of *dvrelish* (**Figure 6B**). At 24 hpe, *dvrelish* transcription was significantly reduced in unexposed ticks compared to ticks injected with GFP or PBS only and to ticks that were exposed to *R. rickettsii* (**Figure 6C**). At 48 hpe, siRelish had no impact on *dvrelish* transcription in the unexposed ticks, but a significant reduction in transcription was observed in the siRelish-treated ticks exposed to *R. rickettsii* (**Figure 6D**). These observations suggest that siRelish had an effect through 24 h and the exposure to *R. rickettsii* had a variable impact on the transcription of *dvrelish* expression in ticks. When examining rickettsial load in ticks exposed to either siRelish or controls, no differences in rickettsial load was observed through 24 h, while *dvrelish* knockdown resulted in increased *R. rickettsii* load at 48 hpe (**Figure 6E**). Together, these results showed that when *dvrelish* transcription is unchanged, rickettsial loads are also unchanged; however, if *dvrelish* is lower, rickettsial loads are greater.

## Discussion

Ticks serve as vectors for a number of obligate intracellular bacteria. Within the Rickettsiales, members of the SFG *Rickettsia* are unique by employing ticks as both reservoir hosts and vectors to transit to vertebrate hosts (Sonenshine and Macaluso, 2017). The progression of infection is not fully characterized for SFG *Rickettsia* in ticks. If ingested while a female tick takes a bloodmeal, rickettsiae infect the midgut and then transit to other tick tissues, including the hemolymph, salivary glands, and ovaries. As tick hemocytes are free floating in tick hemolymph, it is likely that infection occurs for cells independent of the cell-to-cell spread observed in monoculture. If infection of the ovaries or salivary glands occurs, SFG *Rickettsia* can be transmitted to progeny (vertical) or new hosts (horizontal) (Sonenshine and Macaluso, 2017). For *Dermacentor ticks*, infection with *R. rickettsii* can result in reduced tick fitness (Niebylski et al., 1999; Schumacher et al., 2016; Harris et al., 2017). Furthermore, a differential transcriptional response, including immune components, is activated in ticks when infected with SFG *Rickettsia* (Mulenga et al., 2003; Macaluso et al., 2003; Sunyakumthorn et al., 2013; Martins et al., 2019; Guizzo et al., 2022).

In the current study, the immune factor Relish-type NF-κB was examined in *D. variabilis.* The successful isolation of a full-length transcript of *dvrelish* identified a putative transcription factor responsive to Gram-negative bacteria, as described in other arthropods (Hetru and Hoffmann, 2009). Because there is no genome sequenced for *D. variabilis*, RACE was employed to fully characterize the molecule given the limited transcriptomes with evidence of partial NF-kB transcripts. Identifying the full transcript and its untranslated regions may also provide evidence of regulatory mechanisms for future studies. The *dvrelish* transcript is produced at a low level and only the cDNA from *Rickettsia*-infected *D. variabilis* contained detectable amounts of *dvrelish* transcript. This was not unexpected as infection in arthropods induces the increased transcription of immune-related genes, including those encoding NF-κB proteins (Meister et al., 2005; Tanaka et al., 2007; Antonova et al., 2009). To obtain full-length transcript, RACE-PCR was employed as has been done for other *Dermacentor* genes (Mulenga et al., 2004; Sunyakumthorn et al., 2012; Petchampai et al., 2014). Within the sequence, conserved domain searches identified the RHD of *dvrelish* as Relish type, as opposed to Dorsal/Dif-type. While the nucleotide and amino acid identities are low compared to other model arthropods, the amino acids responsible for the protein function of Relish-type NF-κB are highly conserved in the RHD, IPT domain, and ankyrin repeats. In *Drosophila*, the conserved domains of NF-κB allow for binding to DNA, dimerization, and the binding of the encoded ankyrin repeats for sequestration in the cytoplasm of cells (Hetru and Hoffmann, 2009). While an NF-κB protein has been annotated in the *I. scapularis* genome, the sequence does not contain a critical canonical domain of the ankyrin repeats (Smith and Pal, 2014; Rosa et al., 2016). Dorsal-type NF-κB proteins are unique in having a separate inhibitory protein that sequesters the protein in the cytoplasm; however, Relish proteins have encoded ankyrin repeats, which act as the inhibitory domain (Hetru and Hoffmann, 2009). Once activated, the inhibitory N-terminal portion of the protein is cleaved, the nuclear localization sequence is exposed, and it is translocated into the nucleus of the cell. The presence of encoded ankyrin repeats supports the classification of *dvrelish* as encoding a putative Relish-type NF-κB protein.

To further characterize DvRelish, protein expression was assessed in uninfected, unfed adult female *D. variabilis*. Two protein bands of interest were specifically recognized, and both were analyzed by mass spectrometry. Peptide competition assays confirmed the specificity of the antibody used for detection. The identification of both the full-length DvRelish protein and the cleaved N-terminal DvRelish demonstrated basal levels of expression of DvRelish in tick tissues. Relish-type NF-κB proteins are known to be expressed at basal levels in model arthropods, and after immune activation, both mRNA transcription and protein translation are increased (Meister et al., 2005; Fan et al., 2008; Tanji et al., 2010). Previous reports also suggest that immune responsive transcription factors are active in ticks infected with SFG *Rickettsia* (Mulenga et al., 2003; Macaluso et al., 2003; Sunyakumthorn et al., 2013; Martins et al., 2019; Guizzo et al., 2022). The influence of signaling mechanisms controlling tissue-specific and bacteria-specific immune responses and how these responses mediate the dissemination of SFG *Rickettsia* in their tick vectors require further study.

Next, we examined the activation mechanisms associated with DvRelish. The canonical Relish of *Drosophila* is a modular protein consisting of two domains, RHD and IκB. The RHD domain enters the nucleus and activates the expression of AMP genes as active transcriptional mediators (Stoven et al., 2003; Meister et al., 2005). Relish was described to be similar to mammalian NF-κB molecules, p105 and p100, which contain the RHD domain (Dushay et al., 1996). Furthermore, activation of Relish can induce immunity and embryogenesis, suggesting that similar proteins were present in primordial immune systems (Dushay et al., 1996). However, Dif, Dorsal, and Relish can form different homo- and heterodimeric combinations that have distinct DNA-binding affinities (Han and Ip, 1999). In the *Drosophila* IMD pathway, after the phosphorylation of Relish, the Rel moiety migrates to the nucleus where it regulates the expression of genes encoding AMPs and many other proteins (De Gregorio et al., 2002; Ferrandon et al., 2007). To assess the DNA binding of DvRelish, we generated rDvRHD for use in an EMSA. Binding of DvRelish RHD to κB motifs suggests that DvRelish is a transcription factor and activates κB motifs to regulate the expression of immune-regulated genes. A direct association between DvRelish binding to κB motifs and abundance of tick AMPs was not investigated in the current study but should be examined.

Because tick hemocytes are important in AMP production (Johns et al., 2001; Hynes et al., 2008), we explored differential expression of DvRelish during bacterial insult. We utilized *P. aeruginosa* as a non-tick-associated bacteria to contrast the tick-borne *R. rickettsii*. Qualitative analysis indicated increased expression of DvRelish in hemocytes from *R. rickettsii*-exposed ticks, compared to *P. aeruginosa*-exposed ticks at 1 hpi. Several variables may account for observed differences including time and origin of stimulation. The expression of Relish-type NF-κB proteins has been previously visualized in cultured *Drosophila* cells indicating nuclear translocation in as little as 10 min post-stimulation with *E. coli* peptidoglycan (PGN) (Stoven et al., 2003). As purified PGN of Gram-positive and Gram-negative bacteria has been shown to elicit AMP production via NF-κB signaling (Hedengren-Olcott et al., 2004), the variance in nuclear translocation of DvRelish in *D. variabilis* hemocytes may be due to differential patterns of induction of NF-κB proteins in ticks as compared to other arthropods. While further analyses including confirmation that hemocytes were infected with bacteria is needed, these results suggest that *R. rickettsii* can induce the expression of Relish-type NF-κB in *D. variabilis* hemocytes.

In arthropods, the role of Relish has been characterized using Relish deletion mutants and resulted in increased host susceptibility to bacterial and fungal infection (Hedengren et al., 1999). In the current study, we hypothesized that if DvRelish is critical for the tick innate immune response to infection, silencing the expression of *dvrelish* gene would increase the number of *R. rickettsii* in the ticks. First, the expression of *dvrelish* was confirmed to be significantly reduced in *D. variabilis* with 500 ng of siRNA at 6 h post-injection. Then, to test the hypothesis, siRNA-treated ticks were exposed to *R. rickettsii* and *dvrelish* transcription and rickettsial load were assessed over 48 h. At 48 h post-exposure, there was a significant decrease in *dvrelish* transcription in the treated groups, compared to no siRNA or the non-specific GFP-siRNA control. The reduced *dvrelish* transcription corresponded with increased rickettsial load at 48 h compared to control treatments. The results are consistent with what has been reported for *Anaplasma marginale* infection in *Rhipicephalus microplus*, where RNA-mediated silencing of Relish was associated with significantly greater *A. marginale* loads (Capelli-Peixoto et al., 2017). Overall, the data suggest an inverse relationship between Relish function and rickettsial loads.

The tick immune response and vector interactions with SFG *Rickettsia* have been recently reviewed (Fogaça et al., 2021; Kim, 2022). In the current study, a full-length *dvrelish* transcript was isolated and partially characterized in the tick *D. variabilis*. Further study is needed to examine the signaling that induces DvRelish and the tissue-associated immune molecules that are produced as a result of activation. For example, in *D. andersoni*, blocking IRE1α, an upstream inducer of Relish, led to increased bacterial loads in tick midgut and salivary glands (Sidak-Loftis et al., 2022). While *A. marginale* infection did not induce changes in Relish transcription in *R. microplus* midguts and salivary glands, knockdown of Relish was associated with a decrease in the AMP microplusin and an increase in bacterial loads in these tissues. The tick species and tissue-specific nature are intriguing as it has been reported that basal levels of AMPs are present in the tick midgut (Guizzo et al., 2024), whereas in *Amblyomma maculatum*, infection with *Rickettsia parkeri* induces transcription of more than 11,000 immune-related genes, including Relish, in tick hemocytes (Adegoke et al., 2023). The current study provides novel insight into a unique tick–pathogen system that can serve as a basis to better understand how SFG *Rickettsia* navigate the tick immune response to persist within vector populations.

## Data Availability

The datasets presented in this study can be found in online repositories. The names of the repository/repositories and accession number(s) can be found below: https://www.ncbi.nlm.nih.gov/genbank/, KJ484815.

## Supplementary material

Supplementary Figure 1 Expression of DvRelish Rel homology domain (RHD) in transfected HEK293T cells. HEK293T cells transfected with DvRelish-pAcGFP. The transfected cells were visualized under fluorescence microscope.
