# Supplementary figures and images for: Identification and characterization of a Relish-type NF-κB, DvRelish, in Dermacentor variabilis in response to Rickettsia rickettsii infection
Source: Front Cell Infect Microbiol. 2024 Dec 16;14:1494450. doi: 10.3389/fcimb.2024.1494450 (PMC11682715; doi:10.3389/fcimb.2024.1494450)

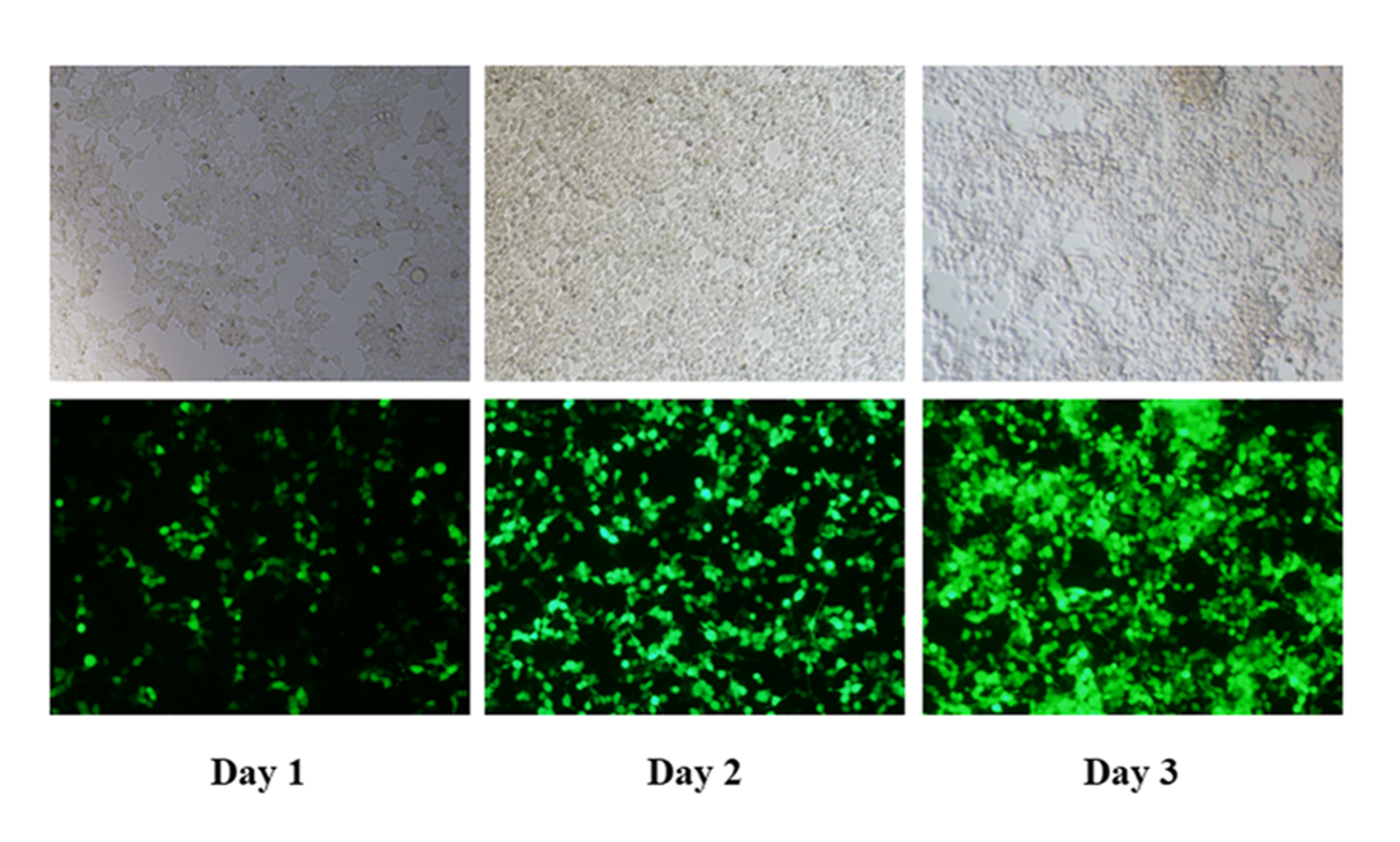

Supplement: Supplementary Figure 1 — Expression of DvRelish Rel homology domain (RHD) in transfected HEK293T cells. HEK293T cells transfected with DvRelish-pAcGFP. The transfected cells were visualized under fluorescence microscope. [file Image1.tif]
